# Isolation and Identification of Pathogens Associated with Fruit Rot of *Tamarindus* *indica* L. and Screening for Their Biocontrol Agents

**DOI:** 10.3390/microorganisms14061300

**Published:** 2026-06-09

**Authors:** Haiwen Wang, Yuxuan Zhai, Jiahui Zang, Junli Feng, Xiaorui Zhang, Xu Qiao, Tingting Dai

**Affiliations:** 1 Co-Innovation Center for the Sustainable Forestry in Southern China, Nanjing Forestry University, Nanjing 210037, China; 13865744264@163.com (H.W.); tyzhaiyuxuan@163.com (Y.Z.); zjh20011009@163.com (J.Z.); 15619215903@163.com (J.F.); 17761709897@163.com (X.Z.); 2 Academician Cao Fuliang’s Workstation, Suining 221200, China; 1749909662@163.com

**Keywords:** *Tamarindus indica* L., pathogen, *Botryosphaeria fabicerciana*, *Bacillus velezensis*, genome analysis

## Abstract

*Tamarindus indica* L., a key economic tree species in tropical regions, suffers severely from postharvest decay. From 2023 to 2025, disease fruits exhibiting pericarp softening, pulp browning, and sticky exudates were collected in Yunnan, China. Pathogenicity tests following Koch’s postulates, combined with morphological characterization and phylogenetic analyses of the internal transcribed spacer (ITS), translation elongation factor 1-alpha (TEF 1α), and beta-tubulin (TUB) gene regions, identified the causal pathogen as *Botryosphaeria fabicerciana* (isolates ZWML-06, ZWML-44, ZWML-17). This is the first report of this postharvest disease on tamarind in Yunnan, filling an etiological gap. Additionally, an endophytic bacterium, designated BV-1, was isolated from asymptomatic pulp tissues. Whole-genome sequencing and phylogenetic analysis identified it as *Bacillus velezensis*. Strain BV-1 exhibited strong in vitro antagonistic activity against the pathogen, indicating promising biocontrol potential. Functional annotation revealed that BV-1 possesses a complex genetic system with developed transporter systems; its core metabolic network is dominated by nitrogen metabolism and redox processes, suggesting a potential “multi-target” antimicrobial mechanism. This study provides a theoretical basis and novel resources for the green control of postharvest diseases in tamarind.

## 1. Introduction

*Tamarindus indica* L. (Tamarind), a tree in the Fabaceae family, produces an economically important pod-like fruit [1]. Native to tropical Africa, Tamarind has been widely introduced into tropical and subtropical regions worldwide [2] and is valued for its ecological adaptability [3] and diverse economic potential [4]. The pulp of tamarind fruit contains organic acids, polysaccharides, and antioxidants and holds considerable promise for applications in food, medicine, and health products [5]. For example, tamarind seed gum, which is extracted from the seeds, exhibits excellent rheological properties and is extensively used in the food and cosmetic industries [6]. In China, Tamarind is mainly distributed in dry, hot valleys in the Yunnan, Hainan, and Guangxi provinces, where it is a characteristic forest tree and pioneer species that promotes soil and water conservation [7]. In addition, Tamarind occupies a significant position in the national strategy for developing green food industries.

Yunnan Province is the principal growing region for Tamarind in China, with plantation-scale and industrial benefits ranking among the highest nationwide [8]. Nevertheless, intensification of cultivation and global climate change have increasingly exposed orchards to disease, which has become a key constraint on sustainable industrial development [9]. Current research on Tamarind diseases has focused largely on foliar pathogens, including leaf spot caused by *Bartalinia robillardiodes* (Flaviano Tassi) [10], powdery mildew caused by *Oidium tamarindi* (J.M. Yen) [11], and sooty blotch caused by *Meliola tamarindi* (H. Sydow & P. Sydow) [12]. Etiological studies of postharvest fruit rot remain uncommon. Field surveys have indicated that postharvest rates of decay of tamarind fruit frequently reach 8–22%, with symptoms including shell softening, pulp browning, and the exudation of viscous fluid [13]. Postharvest decay results in substantial economic losses, but causal pathogens, infection mechanisms, and strategies for disease control remain poorly understood.

Fungi of the genus *Botryosphaeria* are important pathogens of numerous woody plants and are distributed globally [14]. These fungi infect forest trees and fruit crops and cause dieback, cankers [15], and fruit rot [16], which may lead to severe agricultural and forestry losses. The pathogenic mechanisms of Botryosphaeria fungi are complex and commonly involve the secretion of multiple cell-wall-degrading enzymes and the capacity to remain latent within hosts until environmental conditions favor rapid symptom expression [17], which poses a considerable challenge for management. In recent years, fungal species in this genus have caused increasing amounts of damage in tropical and subtropical regions [18]. Among them, *B. fabicerciana* infects a broad range of hosts and has been receiving increased attention for its agricultural significance. However, reports of diseases caused by this fungus remain confined to common hosts, and systematic investigation of its infection capacity and pathogenic traits in other crops is lacking. To date, no investigation of *B. fabicerciana* has occurred in relation to Tamarind fruit rot. Clarifying the pathogenicity and disease characteristics of *B. fabicerciana* in tamarind fruit is essential for understanding the influence of host range and obtaining an early warning of potential disease risks.

With the increasing awareness of the economic and ecological impacts of these pathogens, ongoing research and development of control strategies are essential to safeguard plant health and productivity worldwide. The reliance on synthetic fungicides, while effective, poses significant risks to environmental sustainability and human health due to toxic residues and the emergence of fungicide-resistant strains [19]. Consequently, the exploitation of microbial biocontrol agents presents an excellent alternative, offering eco-friendly and sustainable disease management solutions. In the context of tamarind production, *B. fabicerciana* has emerged as a devastating pathogen causing severe postharvest fruit rot, yet effective control measures remain scarce. During our investigation of tamarind orchards in Yunnan Province from 2023 to 2025, widespread fruit rot was observed, leading to substantial economic losses and threatening the viability of the local industry. Given the limitations of conventional chemical control, there is an urgent need to explore endophytic bacteria microbes that reside asymptomatically within plant tissues as potential biocontrol candidates. These endophytes can act as systemic protectants, directly antagonizing pathogens through competitive exclusion, antibiosis, or induced resistance [20]. Therefore, *B. fabicerciana* was selected as the target pathogen for the screening of biocontrol bacteria in this study.

The primary objective of this study was to elucidate the etiology of postharvest fruit rot in tamarind and to develop a sustainable biocontrol strategy based on antagonistic endophytic bacteria. To achieve this overarching goal, the following specific aims were addressed: (1) to isolate and identify the causal pathogen using integrated morphological characterization and multi-gene phylogenetic analysis; (2) to fulfill Koch’s postulates and confirm the pathogenicity of the isolated strain on healthy tamarind fruits; (3) to screen and isolate endophytic bacteria from asymptomatic, healthy tamarind tissues for antagonistic activity against the identified pathogen; and (4) to perform genomic characterization of a promising antagonistic strain and to decipher its potential antifungal mechanisms.

## 2. Materials and Methods

### 2.1. Disease Survey, Sampling and Isolation

Twenty symptomatic tamarind fruits were collected from Maoduoli Estate (101°48′31.60″ E, 23°45′24.34″ N; cultivation area approximately 213.3 ha) in Yunnan Province, China, for subsequent tissue isolation. Symptoms on infected tamarind fruits were photographed and archived using a Canon EOS R50 camera(Canon Inc., Tokyo, Japan).

Pathogens were isolated from symptomatic tamarind fruit by the tissue isolation method. Symptomatic fruits were rinsed under tap water for 10 min, surface-dried with filter paper, and then transferred to a sterile clean bench. Using a sterile scalpel, tissue from the junction of diseased and healthy areas was excised. The tissue was cut into 3 × 3 mm blocks with sterile scissors and placed on a sterile sieve. The tissue was then immersed in 75% ethanol for 30 s, in 1% sodium hypochlorite (NaClO) solution for 90 s, and finally rinsed three times with sterile water for 1–2 min per rinse [21]. Sterile forceps were used to transfer tissue blocks onto sterile filter paper to remove excess moisture. Another pair of sterile forceps was used to place the blocks onto PDA plates (90 mm), which were incubated in the dark at 25 °C for 5–8 days. Hyphal tips from colony margins were transferred with a sterile needle to fresh PDA for purification and preservation. Plates were kept in the dark at 25 °C for 3 days to obtain pure strains [22]. The resulting isolates were purified and stored at 4 °C in 50% glycerol.

### 2.2. Pathogenicity Tests

Healthy asymptomatic fruits were rinsed with sterile water and then immersed in 1 L of aqueous solution containing 16 mL ethanol, 16 mL NaClO, and 0.05 mL Tween 20 for 4 min. Fruits were removed, air-dried on the laboratory bench, and further dried on a ventilated clean bench before inoculation [23].

Mycelial plugs (5 mm in diameter) cultured for 5 days were inoculated onto healthy, unwounded tamarind fruits. Sterile PDA plugs of an equal size were inoculated onto controls (CK). Fruits were placed in sealed plastic containers lined with sterile filter paper and supplied with sterile water at the base. The containers were maintained in a light incubator at 25 °C with 85% relative humidity, a photon flux density 30 μmol m^−2^ s^−1^, and a 16 h photoperiod. Lesion development was monitored and documented over a period of ten days, during which the progression of disease incited by the fungal plugs on the tamarind fruits was closely tracked and photographed [24]. After the appearance of symptoms, re-isolation was performed for comparison with the inoculated strain to complete Koch’s postulates.

### 2.3. Morphological Identification of the Pathogen

Five-millimeter-diameter plugs of pathogenic strains were placed at the center of freshly prepared PDA and incubated at 25 °C for 3–5 d. Colony color, morphology, and texture were observed and recorded. Strains were then cultured in the dark at 25 °C for two months. Stromata were examined using a SteReo Discovery.V20 stereomicroscope (Carl Zeiss MicroscopyGmbH, Jena, Germany). Pycnidia on stromata were punctured with a sterile inoculation needle, diluted with sterile water, and hyphae and spores were imaged with an Axio Scope 5 fluorescence microscope (Carl Zeiss Microscopy, GmbH).

### 2.4. Molecular Identification of the Pathogen

Fungal mycelium was grown in potato dextrose broth (PDB) for 5 d and then surface-dried with sterile filter paper and ground to powder in liquid nitrogen. Genomic DNA (gDNA) was extracted using the CTAB method [25], electrophoresed to assess quality, and stored at −20 °C.

All extracted gDNA samples were used for amplification of the ITS region [26]. Amplification products were sequenced at Shanghai Jieli Biotechnology Co., Ltd. (Shanghai, China) Sequences were compared against the National Center for Biotechnology Information (NCBI) database to preliminarily assign species identity. The relevant literature was consulted, and primers for β-tubulin (TUB) [27] and translation elongation factor 1-alpha (TEF-1α) [28] were selected for the amplification of corresponding gene fragments (Table 1). Primers were synthesized by GenScript (Nanjing, China). Primer sequences are listed in Table 1. PCR reactions (50 μL total volume) contained 19 μL ddH_2_O, 2 μL of each primer, 25 μL Taq polymerase mix, and 2 μL DNA template [29]. Thermal cycling conditions are provided in Table 2. The resulting amplicons were resolved by agarose gel electrophoresis on 1.5% agarose gels stained with GelRed (Biotium, Hayward, CA, USA) and visualized under appropriate illumination. The outcomes were verified by comparison with sequencing data, and homologous sequences were identified through using BLAST (2.17.0) on the NCBI database to confirm the identity of the pathogen.

Nuclear gene sequences from the ITS, TEF1, and TUB regions, as well as mitochondrial gene sequences used for phylogenetic tree construction in this study, were obtained from GenBank. For sequences generated in this work, BioEdit v7.7.1.0 was used to check for ambiguous bases, and any misaligned bases were corrected. Combined with target genes, multiple sequence alignment was performed with MAFFT v7.3.13 [30], followed by trimming of excessive regions with trimAI; manual adjustment was applied when necessary. Multigene sequences were concatenated into a nuclear gene dataset with PhyloSuite^®^ v1.2.3 [31].

Phylogenetic trees were inferred using the maximum likelihood (ML) and Bayesian inference (BI) frameworks and were implemented with IQ-TREE v1.6.12 [32] and MrBayes v3.2.7 [33], respectively. PartitionFinder 2 [34] and MrModeltest v2.4 [35] were used to select the best-fit substitution models for tree construction. The resulting trees were visualized and annotated with iTOL.

### 2.5. Isolation of Endophytic Bacteria from Tamarind Fruits

Symptom-free tissues were excised from infected tamarind fruits using sterile forceps, placed on a sterile sieve, and subjected to a series of surface sterilization treatments: first immersed in 75% ethanol for 30 s, then transferred to a 1% sodium hypochlorite (NaClO) solution for 90 s, followed by rinsing with sterile water three times (1–2 min per rinse). To verify the efficacy of external sterilization, the final rinse was spread on LB medium and incubated at 30 °C for 48 h; the results showed no bacterial contamination in the samples. Subsequently, 10 sterilized tissue pieces were placed in a sterilized mortar containing 1 mL of sterile water and thoroughly ground into a homogenate. The homogenate was evenly spread on the surface of LB agar plates using the spread plate method, then incubated at 30 °C for 48 h to observe single colony formation. Suspected single colonies were picked and further purified by streak plate method. After additional incubation at 30 °C for 48 h, the purified strains were transferred to fresh LB medium and stored at 4 °C for future use [36].

### 2.6. Screening of Endophytic Bacteria from Healthy Tamarind Fruits

A four-point plate standoff method was selected to screen for bacteria with antagonistic effects against pathogens causing fruit rot of tamarind [37]. Plugs were obtained with a sterilized puncher (6 mm diameter) from the edge of pathogen colonies grown for 3 days and transferred to the center of fresh PDA medium. A total of 15 endophytic bacterial isolates were individually co-cultured with the pathogen at a distance of 1.5 cm. Uninoculated control groups were maintained under identical conditions. All experiments were performed in triplicate and incubated at 28 °C for 4 days.

### 2.7. Molecular Identification of Endophytic Bacteria

Following cultivation in LB broth (30 °C, 160 rpm, 12 h), endophytic bacteria were pelletized via centrifugation (12,000× *g*, 4 °C, 5 min) and resuspended in 100 µL ddH_2_O. Genomic DNA was released using two cycles of freeze–thaw treatment (100 °C for 5 min, followed by −20 °C for 10 min) and cleared by centrifugation. The 16S rDNA region was amplified with primers 27F/1492R [38] using a standard PCR mix (Table 3) (Takara Bio, Beijing, China) and sequenced commercially (Shanghai Jieli Biotech). Raw sequences were assembled and aligned using BioEdit v7.0.9.1 [39] and Clustal W (v2.1). Phylogenetic reconstruction was performed using the ML method in IQ-TREE v1.6.8 based on the best-fit model selected by ModelFinder [40], with 1,000,000 bootstrap replicates. The resulting tree was visualized in FigTree v1.1.4.

### 2.8. Genome Sequencing

The genomic DNA was extracted by using the Cetyltrimethyl Ammonium Bromide (CTAB) method with minor modification, and then the DNA concentration, quality and integrity were determined by using a Qubit Flurometer (Thermo Fisher Scientific, Waltham, MA, USA) and a NanoDrop Spectrophotometer (Thermo Scientific, Waltham, MA, USA). Sequencing libraries were generated using the TruSeq DNA Sample Preparation Kit (Illumina, San Diego, CA, USA) and the Template Prep Kit (Pacific Biosciences, Menlo Park, CA, USA). The genome sequencing was then performed by Personal Biotechnology Company (Shanghai, China) using the Illumina Novaseq platform. Data assembly was proceeded with after adapter contamination removing and data filtering by using AdapterRemoval (v2.3.4) and SOAPec (v2.0). The filtered reads were assembled by SPAdes and A5-miseq to construct scaffolds and contigs. Finally, the genome sequence was acquired after the rectification by using Pilon software. Genome function elements prediction included the prediction of coding-gene, non-coding RNA and clustered regularly interspaced short palindromic repeats (CRISPRs). Gene prediction was performed by GeneMarkS v4.32. tRNAscan-SE, while Barrnap (version 0.9) and Rfam were used to find tRNA, rRNA and other ncRNA, respectively. CRISPR (v1.2) finder was identified by CRISPR recognition tool. Repeat sequence was analyzed using RepeatModeler (v23.06) software. RepBase database was used to predict sequences similar to known repeat sequences. TRF software (version 4.10.0) was used for tandem repeat prediction. For subsystem, PhiSpy (v4.2.21) and IslandViewer 4 software were respectively used to predict the prophages and genomics islands. Subsequently, the VFDB (Virulence Factors of Pathogenic Bacteria) database and CARD (The Comprehensive Antibiotic Resistance) database were used to retrieve the pathogenicity genes and antibiotic resistance genes, respectively. The CAZy (Carbohydrate-Active enzymes) database was used to predict Carbohydrate-Active enzymes and the MvirDB database was used to predict protein toxins and virulence.

Function annotation was completed by blast search against different databases, including NR (Non-Redundant Protein Database), GO (Gene Ontology), KEGG (Kyoto Encycolpedia of Gene and Genomes), COG (Cluster of Orthologous Groups of proteins, Swissprot, Pathogen–Host Interactions Database (PHI), Transporter Classification Database (TCDB), Pfam and MEROPS.

## 3. Results

### 3.1. Disease Symptoms and Fungal Isolation

**Figure 1:**
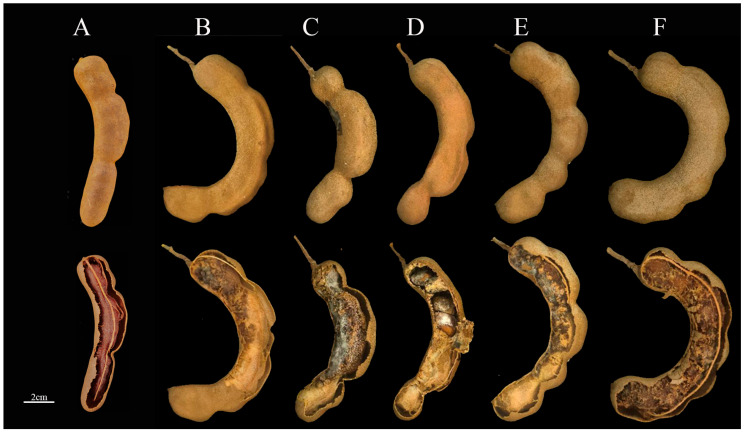
Tamarind fruits. (**A**) Healthy control (CK) fruit. (**B**–**F**) Diseased fruits. The upper row shows intact unpeeled fruits, and the lower row shows the corresponding peeled fruits.

Unpeeled tamarind pods retained their typical crescent shape, and structural integrity was largely preserved (Figure 1A). Some pods exhibited abnormal coloration, and we observed a gradual transition from a localized or extensive light brown color to a dark brown color, which was accompanied by reduced epidermal glossiness and occasional fine shriveling or uneven pigmentation. Infected pulp presented soft rot or maceration and appeared visually spongy and collapsed. In some regions, tissue disintegration led to cavitation or signs of exudate release, with indistinct boundaries between diseased and healthy tissues. At the center of affected areas, yellowish-brown discoloration was covered with white fungal mycelium that expanded with disease progression. Mycelium and brownish discolored zones on the fruit gradually coalesced into patchy or mass-like patterns. In severe cases, most of the pericarp and surrounding pulp turned dark brown (Figure 1B–F).

### 3.2. Pathogen Isolation and Morphological Observations

**Figure 2:**
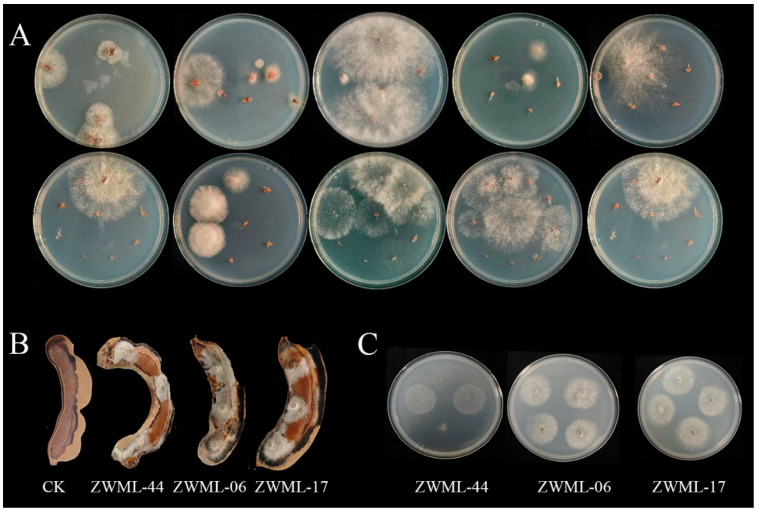
(**A**) Fruit pulp tissue fragments (N = 69) were isolated and cultured on potato dextrose agar (PDA). After 10 days of incubation, ten isolates were obtained. (**B**) Tamarind fruits inoculated with strains ZWML-06, ZWML-44 and ZWML-17 developed mold growth and rot symptoms 10 days after inoculation. CK: control group. (**C**) Re-isolation performed following Koch’s postulates yielded isolates with morphological features identical to those of the original strains ZWML-06, ZWML-44 and ZWML-17.

Following the collection of symptomatic tamarind fruits in the field, tissue isolation was performed in the laboratory. From these samples at Maoduoli Estate, a total of 69 fruit pulp fragments were isolated. A total of 10 isolates exhibiting identical morphological characteristics were recovered, and three representative strains (ZWML-06, ZWML-44 and ZWML-17) were selected for further analysis (Figure 2A), including morphological characterization, phylogenetic analysis, and pathogenicity assessment. Strains were inoculated onto tamarind fruits and observed for 10 days. All three strains produced symptoms on tamarind fruits that closely resembled those observed in the field. Healthy tamarind fruits exhibited a uniform coloration ranging from light to dark brown and possessed a smooth, firm pericarp (Figure 2B). On pathogen-inoculated fruits, irregular brown to grayish-brown lesions appeared, which were small at the onset but grew larger and merged as the disease progressed. Lesion surfaces were often covered with abnormal mold growth. Internal tissue necrosis and water loss caused fruit wrinkling and softness. The pulp lost firmness, and fruits became entirely soft-rotted in some cases. Pathogen re-isolation from the diseased fruits yielded isolates that matched the inoculated strains in morphology and pathogenic symptoms (Figure 2C). According to Koch’s postulates, strains ZWML-06, ZWML-44 and ZWML-17 were identified as the causative agents of tamarind fruit rot.

**Figure 3:**
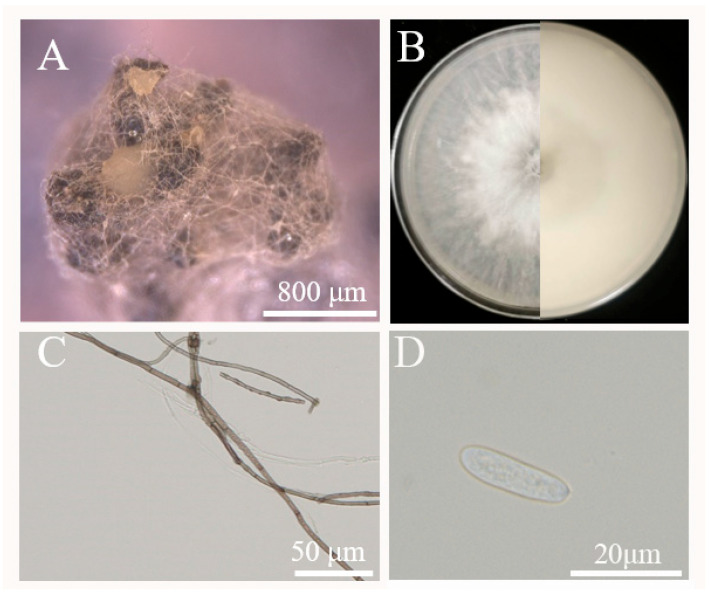
Morphological characteristics of *B. fabicerciana* at different scales. (**A**) Morphological characteristics of *B. fabicerciana* at different scales. (**A**) Stroma (800× magnification) exhibiting a complex reticulate structure with variation in coloration. (**B**) Colony morphology on potato dextrose agar (PDA) after 5 days, as observed from obverse and reverse sides. The colony diameter was approximately 9 cm, and the colony was radially spreading with a dry surface and prominent radial striations. (**C**) Hyphal morphology (50× magnification) showing branched hyphae and septate structures. (**D**) Conidial morphology (10× magnification) showing ellipsoidal to elongated-ellipsoidal with smooth surfaces.

On PDA plates, strain ZWML-06 produced a white mycelium after 5 d of incubation, which turned black after 10 days. Colonies grew uniformly and had a vigorous, cottony mycelium. Under a stereomicroscope, multiple orange-yellow pycnidia were observed on the stromata of strain ZWML-06 (Figure 3A,B). These pycnidia were ovoid, spherical, or subspherical in shape. Examination under oil immersion revealed the hyphal morphology and conidia of ZWML-06 (Figure 3C), which enabled the identification of this strain as Botryosphaeria. Hyphae appeared as slender filamentous strands interwoven into a network. Their color was pale and ranged from nearly transparent to light brown, and the hyphae exhibited typical fungal hyphal characteristics. Hyphal diameter was relatively uniform, and some hyphae branched at varying angles. The hyphae were long, which indicates good extensibility. Overall, hyphal morphology was regular; surfaces were smooth, septa were present, and there were no conspicuous appendages or specialized structures. The conidia of Botryosphaeria (Figure 3D) were ellipsoid to elongate-ellipsoid in shape and morphologically uniform. The size of conidia was moderate, individual conidia were hyaline, and their length and width were 20 μm ± 2 μm × 5 μm ± 0.9 μm (Sample = 50). Conidial surfaces appeared smooth. Cell walls were thin and transparent, and the internal contents were faintly visible.

### 3.3. Molecular Phylogeny and Species Verification of Isolated Fungal Strains

**Figure 4:**
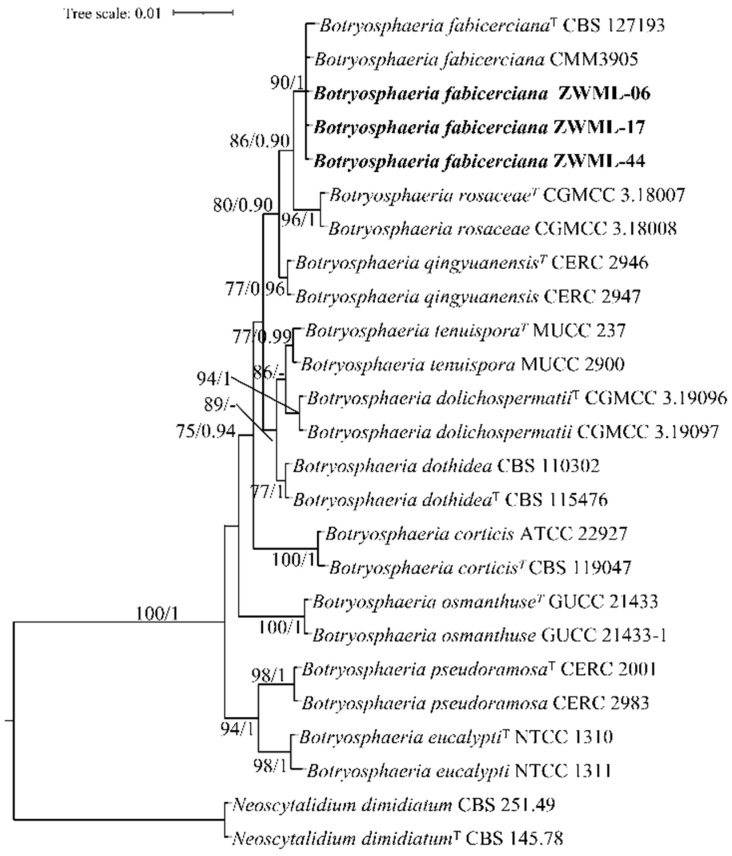
Phylogenetic tree inferred from concatenated sequences from the ITS, TEF1, and TUB regions was generated using maximum likelihood analysis. Bootstrap values greater than 50% (1000 replicates) are shown at nodes. Bar = 0.01 substitutions per nucleotide position. *Neoscytalidium dimidiatum* CBS 251.49 was used as the outgroup. “T” denotes type strain.The numbers in the figure represent the strain accession numbers, and the bolded items indicate the pathogenic fungi screened and identified in this study.

To confirm the morphological identification at the molecular level, the ITS, TEF1-α, and TUB2 gene sequences of strains ZWML-06, ZWML-44 and ZWML-17 were amplified and sequenced. These sequences have been deposited in the GenBank database, with the following accession numbers assigned to each strain: ZWML-06: PZ118602, PZ166962, PZ166965; ZWML-44: PZ118603, PZ166963, PZ166966; ZWML-17: PZ167946, PZ166964, PZ166967. Alignment results showed that the ITS, TEF1-α, and TUB2 sequences of the isolates shared 90% similarity with those of the type Strain *B. fabicerciana* CBS 127193^T^ (HQ332197, HQ332213, KF779068) and *B. fabicerciana* CMM3905 (JX513642, JX513621). Phylogenetic analysis using maximum likelihood (ML) and Bayesian inference (BI) methods clustered isolates ZWML-06, ZWML-44 and ZWML-17 with *B. fabicerciana* CMM3905 and *B. fabicerciana*^T^ CBS 127,193 in a well-supported clade. The ITS, TEF1-α, and TUB2 sequences of reference isolates were retrieved from GenBank (Table 4), and a phylogenetic tree was constructed (Figure 4).

### 3.4. Screening of Endophytic Bacteria for Biocontrol

**Figure 5:**
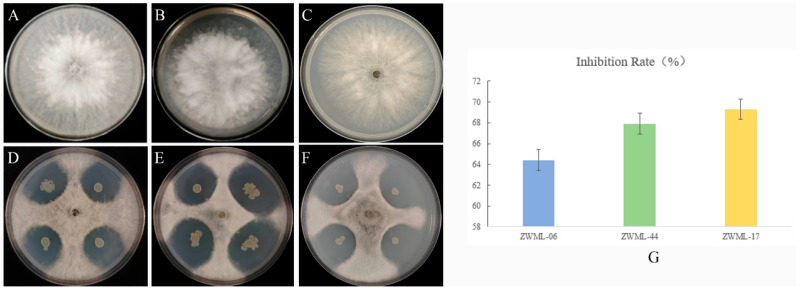
Antifungal efficiency of strain BV-1 against the plant-pathogenic fungus *B. fabicerciana* (strains ZWML-06, ZWML-44, and ZWML-17). (**A**–**C**) show the colony growth of ZWML-06, ZWML-44, and ZWML-17; (**D**–**F**) display the inhibition zones of BV-1 against these three fungi; (**G**) compares the inhibition rates of BV-1 toward ZWML-06, ZWML-44, and ZWML-17.

Fifteen endophytic bacterial strains were isolated from the asymptomatic pulp tissues of diseased tamarind fruits. Of these, only one strain exhibited strong antagonistic activity against the pathogens ZWML-06 (Figure 5A,D), ZWML-17 (Figure 5B,E), and ZWML-44 (Figure 5C,F). This strain, designated as BV-1, significantly inhibited the mycelial growth of all three pathogens on PDA medium (Figure 5G). Quantitative analysis revealed inhibition rates of 64.4%, 67.9%, and 69.3% against ZWML-06, ZWML-17, and ZWML-44, respectively.

### 3.5. Phylogenetic Analyses of Endophytic Bacteria

**Figure 6:**
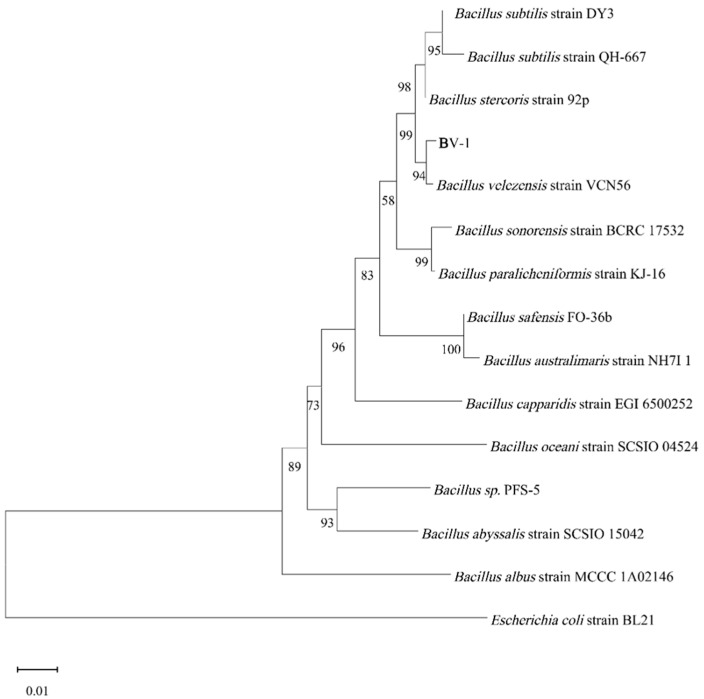
Phylogenetic relationships of *B. velezensis* BV-1 employing Maximum Likelihood (ML) using 16S rRNA sequences, with *Escherichia coli* designated as the outgroup (The numbers in the figure correspond to the strain codes of the isolated strains, and the bolded texts represent the names of antagonistic bacteria against the pathogenic pathogens screened in this study).

The 16S rRNA gene sequence of the endophytic bacterium BV-1 has been deposited in GenBank under the accession number listed in Table 5. BLAST analysis revealed 99.8% sequence identity between BV-1 and *B. velezensis* VCN56 (GenBank accession no. OM349153.1). As anticipated, phylogenetic analysis based on the Maximum Likelihood (ML) algorithm clustered BV-1within the same clade as *B. velezensis*, supported by a bootstrap value of 94% (Figure 6). Integrating multi-locus phylogenetic analysis with morphological characteristics, strain BV-1 was conclusively identified as *B. velezensis*.

### 3.6. Whole-Genome Sequencing of Antagonistic Bacteria

**Figure 7:**
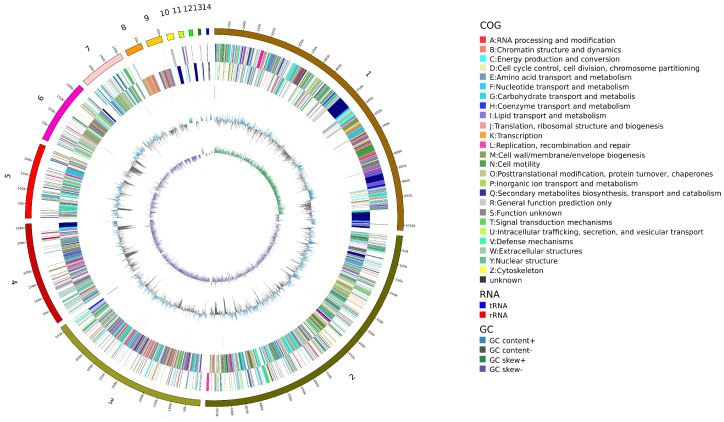
Circular representation of *B. velezensis* BV-1 genome (from the outside—in, they are the karyotype of the chromo some, CDS on the positive and negative strands (different colors indicate different COG (Cluster of Orthologous Groups) classifications of CDS (Coding Sequences), transfer RNA and ribosomal RNA, GC content, and the innermost circle is GC-skew).

**Figure 8:**
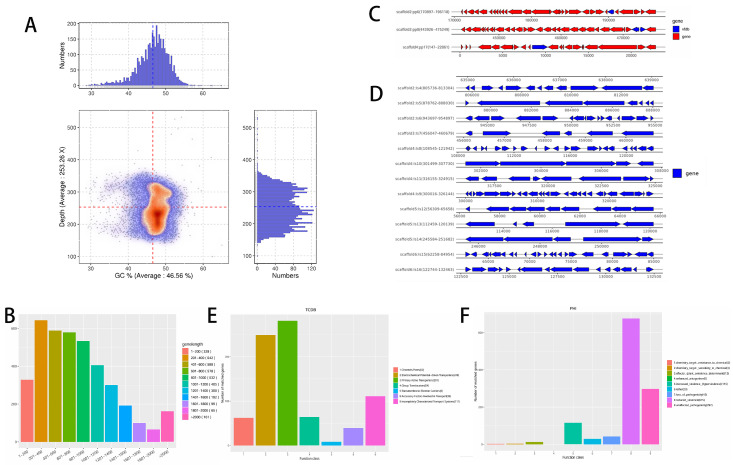
Genomic architecture and functional profiling of antagonistic bacterium BV-1. (**A**) Distribution map of GC_ Depth. (**B**) Gene length distribution map. (**C**) The distribution map of cards and vfs on the prophage. (**D**) Gene island gene distribution map. (**E**) The first-level classification diagram of TCDB functions. (**F**) Distribution map of PHI phenotypic mutation types.

According to the genomic evaluation results, BV-1 possesses a circular chromosome of approximately 3,141,501 bp, with a GC content of 46.56% (Figure 7). The distribution of GC content and sequencing depth of the genome is shown in Figure 8A.

The genomic landscape of antagonistic bacterium BV1 revealed the widespread distribution of antimicrobial resistance genes (*card*) and functional genes, with specific enrichment observed on genomic islands Is6 and Is9. Although these regions harbor pathogenicity/resistance-associated elements, they likely enhance bacterial fitness in complex environments by regulating host metabolic pathways (Figure 8C,D). The Transporter Classification Database (TCDB) analysis (Figure 8E) indicated that strain BV-1 is enriched in transporter protein genes, predominantly categorized under primary active transport (281 genes, Category 3) and electrochemical potential-driven transport (249 genes, Category 2). Furthermore, the Pathogen—Host Interaction (PHI) database annotation (Figure 8F) demonstrated that the majority of genes (675) are associated with reduced virulence, while subsets were identified as neutral (297) or virulence-enhancing (115). These findings suggest that BV-1 employs a sophisticated strategy combining a highly developed transport system for efficient nutrient exchange and a virulence regulation mechanism primarily characterized by attenuation. This dual capability provides a molecular basis for its antifungal antagonism and ecological adaptability.

**Figure 9:**
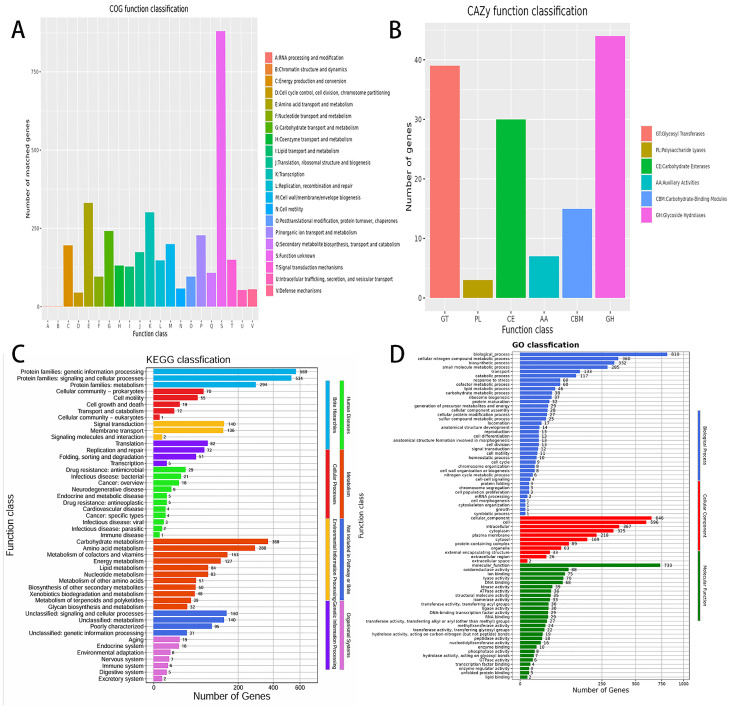
Coding gene annotation results of *B. velezensis* BV-1bacteria. (**A**) COG function classification. (**B**) CAZy function classification. (**C**) KEGG classification. (**D**) GO classification.

Functional annotation of *B. velezensis* BV1 elucidated the core characteristics of its genome. Cluster of Orthologous Genes (COG) classification (Figure 9A) showed that genes with unknown functions (Category S, 900 genes) constituted the largest proportion. However, metabolic functions dominated, including amino acid metabolism (Category E, 300), inorganic ion transport and metabolism (Category P, 250), and carbohydrate metabolism (Category G,250). Additionally, genetic information processing systems, such as transcription (Category K, 300) and translation (Category J, 300), were prominent. Carbohydrate—Active enzymes (CAZy) annotation (Figure 9B) revealed that glycoside hydrolases (GH, 45) and glycosyltransferases (GT, 39) form the core carbohydrate-active modules, supplemented by carbohydrate esterases (CE, 30) and carbohydrate-binding modules (CBM, 15). Kyoto Encyclopedia of Genes and Genomes (KEGG) pathway analysis (Figure 9C) further confirmed amino acid metabolism (368 genes) and carbohydrate metabolism (288 genes) as the central metabolic pillars, supported by energy metabolism (127 genes) and cofactor metabolism (153 genes). Significant enrichment was also observed in signal transduction (140 genes) and membrane transport (136 genes). Gene Ontology (GO) analysis (Figure 9D) highlighted that biological processes were richest in nitrogen compound metabolic processes (810 genes), followed by biosynthetic processes (332 genes). At the molecular level, oxidoreductase activity (733 genes) and ion binding (705 genes) were predominant, with cellular components primarily localized to the cytoplasm (325 genes) and membrane structures (218 genes). In summary, strain BV1 possesses a highly developed nitrogen metabolism and redox system, a comprehensive metabolic network, and significant environmental adaptation potential. The abundance of metabolic functional genes aligns closely with its application profile as a plant growth promoter and biocontrol agent, although a substantial number of genes remain functionally uncharacterized.

## 4. Discussion

*T. indica* is an important species in Yunnan’s plateau-specific agriculture; the tree is ecologically adaptable, and the fruit possesses nutritional and health-promoting value and represents a suitable crop for economic development in dry-hot valley regions [41]. The unique subtropical plateau monsoon climate of Yunnan produces high humidity during the rainy season, and frequent outbreaks of fungal disease pose a serious constraint to sustainable development of the *Tamarind* industry [42,43]. This study provides the first characterization of typical symptoms of tamarind fruit rot (browning of the pulp and exudation of viscous fluid), and we identified *B. fabicerciana* as etiological agent.

To enable the development of effective control strategies, we first isolated and identified three pathogenic strains, ZWML-06, ZWML-44 and ZWML-17, using tissue isolation combined with verification through Koch’s postulates. The symptoms produced by these strains matched those of natural infections. Morphological observation together with concatenated phylogenetic analysis of the ITS, TUB and TEF1 gene regions enabled us to identify our isolates as *B. fabicerciana*. Field surveys indicated that infected fruit shells showed no obvious mechanical damage, which makes symptomatic fruits difficult to recognize by their external appearance. The fungal pathogen may enter through micro-wounds or natural openings such as lenticels during preharvest or harvest and remain latent within the fruit. Upon fruit maturation or under favorable conditions, the fungus becomes active and induces symptom development [44]. *Botryosphaeria* species possess active dispersal capacity and can spread to healthy fruits via wind and rain to continue the infection cycle [45].

Compared with brown rot or dieback symptoms caused by *B. fabicerciana* in mango [16] and eucalyptus [46], we found that the fungus not only induced browning and softening of pulp but also formed grayish-black velvety mycelial layers and exudates on and within the pulp. Unlike in mango or eucalyptus, the enclosed structure of the tamarind shell may promote extremely rapid whole-fruit decay once infection occurs [47]. The dense fibrous composition of tamarind pulp suggests that decay involves complex cell wall degradation processes. Previous work has shown that *Botryosphaeria* secretes pectinases, cellulases, and other cell-wall-degrading enzymes to break down host tissues [48,49].

Although tamarind is rich in organic acids, such as tartaric acid, which generally inhibit microbes, *B. fabicerciana* can colonize tamarind fruit and cause disease in acidic conditions; this is consistent with an acid-tolerant mechanism that was reported in *B. dothidea* [50]. Future research should focus on identifying the molecular mechanisms, particularly the regulatory networks involved in acid stress responses and polysaccharide degradation, underlying the pathogenicity of *B. fabicerciana* in the unique microenvironment of tamarind fruit.

Whole-genome sequencing of the antagonistic endophyte *B. velezensis* BV-1 revealed a genome size of approximately 3.14 Mb with a GC content of 46.56%, consistent with the established genomic range of typical *B. velezensis* strains [51]. A pivotal finding was the significant enrichment of genes associated with “reduced virulence” (675 genes) annotated against the PHI database. This genomic profile contrasts sharply with the repertoire of plant-pathogenic *Bacillus* species, which are predominantly characterized by virulence-related factors [52]. Such architectural features strongly suggest that BV-1 employs a “cryptic” or “host-friendly” colonization strategy, prioritizing competitive exclusion and antibiosis over direct parasitic interactions [53]. This observation aligns with the “multi-target inhibition” hypothesis proposed for *B. velezensis*, wherein broad-spectrum antagonism is achieved through a combinatorial “arsenal” of secondary metabolites rather than reliance on a single potent toxin [54].

Functional annotation provided granular insights into the metabolic basis underlying this “chemical warfare.” Transporter Classification Database (TCDB) analysis revealed significant enrichment of genes related to primary active transport and electrochemical potential-driven transport, indicating that BV-1 possesses highly efficient systems for metabolite export and nutrient acquisition [55,56]. Far from being a passive trait, this implies that BV-1 may outcompete pathogens through the rapid secretion of antifungal compounds [57] or the competitive chelation of limiting nutrients [58]. This genomic signature distinguishes BV-1 from less efficacious biocontrol agents and aligns with the genomic blueprint characteristic of highly competitive environmental *Bacillus* isolates, such as *Bacillus licheniformis* BL1 [59]. Carbohydrate-Active enzyme (CAZy) annotation showed that the BV-1 genome encodes glycoside hydrolases and glycosyltransferases. This enzymatic repertoire closely mirrors that of the reported strain of *B. velezensis*, which has been confirmed to produce cell-wall-degrading enzymes such as cellulases and chitinases [60]. Similarly, another conspecific strain LZH-5 has also been demonstrated to secrete diverse hydrolytic enzymes [61]. These findings indicate that the secretion of cell-wall-degrading enzymes to directly compromise pathogen hyphal structures represents a conserved antagonistic strategy in *B. velezensis*.

Gene Ontology (GO) analysis highlighted a significant enrichment of genes involved in nitrogen compound metabolism and oxidoreductase activity, revealing a distinct metabolic bias toward redox reactions and nitrogen utilization [62]. This metabolic characteristic is particularly notable, suggesting that BV-1 is well-adapted to the acidic and nutritionally heterogeneous microenvironment of tamarind pulp. We hypothesize that this redox-centric metabolism not only fuels the energy-intensive biosynthesis of secondary metabolites [63] but may also modulate local pH or reactive oxygen species levels, thereby indirectly inhibiting pathogen colonization [64].

In summary, genomic data could redefine BV-1 not merely as an “effective antagonist” but as a “genetically pre-assembled” biopesticide. Its potent activity arises from an integrated system encompassing transport, metabolism, and biosynthesis. This genomic foundation provides robust evidence for the further development of BV-1 as a potent bio-preservative for postharvest disease management.

## 5. Conclusions

This study elucidates *B. fabicerciana* as the primary causal agent of postharvest rot in Yunnan tamarind, integrating morphological and multi-gene phylogenetic evidence to resolve its taxonomic status. We further characterized *B. velezensis* BV-1 as a potent endophytic antagonist against this pathogen. Genomic analysis revealed that BV-1 harbors a sophisticated genetic arsenal, featuring highly developed transport systems and a core metabolic network dominated by nitrogen metabolism and redox processes. Notably, the prevalence of genes associated with reduced virulence and Carbohydrate-Active enzymes suggests a “multi-hit” inhibition strategy, likely mediated by antifungal metabolites and cell-wall-degrading enzymes. By linking pathogen etiology with antagonist genomics, this work provides a theoretical foundation for developing eco-friendly means of control derived from bacterial metabolites, offering a sustainable alternative to synthetic fungicides for tamarind postharvest management.

## Figures and Tables

**Table 1:** PCR amplification primer sequences.

Gene Fragment	Primers	Primer DNA Sequence (5′-3′)
ITS	ITS1	TCCGTAGGTGAACCTGCGG
	ITS4	TCCTCCGCTTATTGATATGC
EF1	EF1-688F	CGGTCACTTGATCTACAAGTGC
	EF1-1251R	CCTCGAACTCACCAGTACCG
Bt2	Bt2a	GGTAACCAAATCGGTGCTGCTTTC
	Bt2b	ACCCTCAGTGTAGTGACCCTTGGC

**Table 2:** Programs of PCR.

Gene Fragment	Operation Parameter
ITS	94 °C, 3 min (94 °C, 30 s 56 °C, 30 s 72 °C, 30 s) × 35 72 °C, 10 min
TEF1	94 °C, 3 min (94 °C, 30 s 58 °C, 30 s 72 °C, 45 s) × 35 72 °C, 10 min
Bt2	94 °C, 3 min (94 °C, 30 s 58 °C, 30 s 72 °C, 45 s) × 35 72 °C, 10 min

**Table 3:** Primer sequences and PCR amplification procedures used for the identification of endophytic bacteria.

Primers	Sequence	Operation Parameter
27F	AGAGTTTGATCCTGGCTCAG	94 °C, 3 min; (94 °C, 30 s, 58 °C, 30 s; 72 °C, 15 s) × 30; 72 °C, 10 min
1492R	TACGGYTACCTTGTTACGACTT	

**Table 4:** Species used for phylogenetic analysis, along with their corresponding GenBank accession numbers.

Code	Strain	ITS	TEF1	TUB
ZWML-06	*Botryosphaeria fabicerciana*	PZ118602	PZ166962	PZ166965
ZWML-44	*Botryosphaeria fabicerciana*	PZ118603	PZ166963	PZ166966
ZWML-17	*Botryosphaeria fabicerciana*	PZ167946	PZ166964	PZ166967
ATCC_22927	*Botryosphaeria corticis*	DQ299247	EU673291	EU673108
CBS_119047	*Botryosphaeria corticis^T^*	DQ299245	EU017539	EU673107
CGMCC_3_19097	*Botryosphaeria dolichospermatii*	MH491971	MH491975	MH562328
CGMCC_3_19096	*Botryosphaeria dolichospermatii^T^*	MH491970	MH491974	MH562327
CBS_110302	*Botryosphaeria dothidea*	AY259092	AY573218	EU673106
CBS_115476	*Botryosphaeria dothidea^T^*	AY236949	AY236898	AY236927
NTCC_1311	*Botryosphaeria eucalypti*	PP349868	PP354985	PP354987
NTCC_1310	*Botryosphaeria eucalypti^T^*	PP349867	PP354984	PP354986
CMM3905	*Botryosphaeria fabicerciana*	JX513642	JX513621	-
CBS_127193	*Botryosphaeria fabicerciana^T^*	HQ332197	HQ332213	KF779068
GUCC_21433_1	*Botryosphaeria osmanthuse*	OL854216	OP650907	OP669377
GUCC_21433	*Botryosphaeria osmanthuse^T^*	OL854215	OP650906	OP669376
CERC_2983	*Botryosphaeria pseudoramosa*	KX277992	KX278097	KX278201
CERC_2001	*Botryosphaeria pseudoramosa^T^*	KX277989	KX278094	KX278198
CERC_2947	*Botryosphaeria qingyuanensis*	KX278001	KX278106	KX278210
CERC_2946	*Botryosphaeria qingyuanensis^T^*	KX278000	KX278105	KX278209
CGMCC_3_18008	*Botryosphaeria rosaceae*	KX197075	KX197095	KX197102
CGMCC_3_18007	*Botryosphaeria rosaceae^T^*	KX197074	KX197094	KX197101
MUCC_2900	*Botryosphaeria tenuispora*	LC585276	LC585148	LC585172
MUCC_237	*Botryosphaeria tenuispora^T^*	LC585278	LC585150	LC585174
CBS_145_78	*Neoscytalidium dimidiatum^T^*	KF531816	KF531795	KF531796
CBS_251_49	*Neoscytalidium dimidiatum*	KF531819	KF531797	KF531799

“T” denotes type strain.

**Table 5:** Isolates and sequences used to identify BV-1.

Species	Isolate	GenBank Accession Number
*Bacillus subtilis*	DY3	KY290590
*Bacillus subtilis*	QH-667	MW380652.1
*Bacillus velezensis*	VCN56	OM349135
*Bacillus sonorensis*	BCRC 17532	EF423592.1
*Bacillus paralicheniformis*	KJ-16	KY694465.1
*Bacillus safensis*	FO-36b	MV228825
*Bacilluis australimaris*	NH7I1	MW228046.1
*Bacillus capparidis*	EGI 6500252	KX119423.1
*Bacillus oceani*	SCSIO 04524	KC160501.1
*Bacillus* sp.	PFS-5	JQ966280.1
*Bacillus abyssalis*	SCSIO 15042	JX232168.1
*Bacillus albus*	MCCC 1A02146	MT810121.1
*Escherichia coli*	BL21	AJ605115.1
*Bacillus stercoris*	92P	PQ097724.1
*Bacillus velezensis*	BV-1	PZ321647

## Data Availability

The original contributions presented in this study are included in the article. Further inquiries can be directed to the corresponding author.
